# Supplementary material for: Delay in Diagnosis and Treatment of Primary Bone Tumors during COVID-19 Pandemic in Poland
Source: Cancers (Basel). 2022 Dec 8;14(24):6037. doi: 10.3390/cancers14246037 (PMC9776000; doi:10.3390/cancers14246037)
Supplement: Supplementary file 1 [file cancers-14-06037-s001.zip › cancers-2088792-supplementary.pdf]

The COVID-19 Survey

This is an anonymous survey to examine the impact of the COVID-19 pandemic on the diagnostic and therapeutic process in patients with primary bone tumors. It contains nine closed questions (yes / no questions). It should take up to 5 minutes to complete all of the questions.

The questionnaire was created as a research project carried out at the Department of Orthopaedics, Traumatology and Musculoskeletal Oncology, Pomeranian Medical University.

Thank you in advance for your time and for completing the survey below.

**In the period March 2020 – March 2022…**	**Answer**
1)… were You tested positive for COVID-19?	Yes/No
2)… have you been diagnosed with respiratory failure due to COVID-19?	Yes/No
3)… made the Covid-19 pandemic it difficult for You to access your GP?	Yes/No
4)… made the Covid-19 pandemic it difficult for You to access an orthopedist or oncologist?	Yes/No
5)… made the Covid-19 pandemic it difficult for You to access radiological examinations?	Yes/No
6)… resulted the COVID-19 pandemic in a biopsy being canceled or delayed?	Yes (how long?)/No
7)… resulted the COVID-19 pandemic in the cancellation or delay of the surgery?	Yes (how long?)/No
8)… resulted the COVID-19 pandemic in the cancellation or delay of the chemotherapy?	Yes (how long?)/No
9)… if not for the COVID-19 pandemic, would You visit your physician earlier?	Yes/No

I am aware that participation in the survey is voluntary and anonymous and I agree to participate in the survey by going to the next part of the survey

………………………………………………………………………….

(date and patient's signature)
